# Taste peptides derived from *Stropharia rugosoannulata* fermentation mycelium and molecular docking to the taste receptor T1R1/T1R3

**DOI:** 10.3389/fnut.2022.960218

**Published:** 2022-07-28

**Authors:** Wen Li, Wanchao Chen, Di Wu, Zhong Zhang, Yan Yang

**Affiliations:** Institute of Edible Fungi, Shanghai Academy of Agricultural Sciences, National Engineering Research Center of Edible Fungi, Key Laboratory of Edible Fungi Resources and Utilization (South), Ministry of Agriculture, Shanghai, China

**Keywords:** *Stropharia rugosoannulata* mycelium, taste peptide, taste characteristics, T1R1/T1R3, molecular docking

## Abstract

This study identified the peptides in the fermentation mycelia of *Stropharia rugosoannulata*. The molecular weight of the peptides was below 3,000 Da. Heptapeptides to decapeptides were the main peptides in the fermentation mycelia of *S. rugosoannulata*. More than 50% of the peptides had salty and umami taste characteristics, and the long-chain peptides (decapeptides to 24 peptides) also played an essential role in the pleasant taste characteristics of mycelium. In the salty and umami peptide of *S. rugosoannulata*, the distribution of non-polar hydrophobic amino acids and polar-uncharged amino acids accounted for a relatively high proportion, and the proportion of polar-uncharged amino acids further increased, with the extension of the peptide chain. P, F, I, l, V, G, S, T, and D were the amino acids with a high proportion in the peptides. The taste peptides can bind to more than 60% of the active amino acid residues in the cavity-binding domain of the T1R1/T1R3 receptors. Hydrogen bond interaction was the primary mode of interaction between the peptides and the receptor. The first and second amino acid residues (such as S, V, E, K, G, and A) at the C-terminal and N-terminal of the peptides were easy to bind to T1R1/T1R3 receptors. Asp108, Asn150, Asp147, Glu301, Asp219, Asp243, Glu70, Asp218 in T1R1, and Glu45, Glu148, Glu301, Glu48, and Ala46 in TIR3 were the key active amino acid sites of taste peptides binding to T1R1/T1R3 receptors.

## Introduction

*Stropharia rugosoannulata* is rich in protein, peptides, and amino acids. It is an edible fungus with a unique taste and high nutritional value. Its protein content is 30%−50% dry weight, higher than most edible fungi and vegetables. It is an essential source of high-quality raw materials and protein and can compensate for the disadvantage of single protein varieties in the plant protein resource market (https://mycorena.com/fungi).

It is an effective way to quickly obtain mycelium protein (intermediate) and important secondary metabolites through liquid fermentation by regulating the enrichment of nutrients and bioactive components in the mycelium (1). Liquid fermentation technology can shorten the time of mushroom culture and obtain the mycelium and its active substances of *S. rugosoannulata*. It has unparalleled advantages in developing nutritional and health products and natural taste-based materials. Our previous studies have found that the peptide content in the fermentation mycelium of *S. rugosoannulata* accounted for 60%−70% of the protein content in the mycelium, and the peptide content in the fruiting body accounted for 22%−28% of the protein content in the fruiting body. The fermentation mycelium of *S. rugosoannulata* had advantages in peptide synthesis. As the primary metabolite in the fermentation mycelium of *S. rugosoannulata*, peptides give the mycelium a pleasant taste and a full mouth feeling (2). At the same time, the mycelium showed good biological activity. The mycelium had an inhibition rate of more than 95% on the angiotensin-converting enzyme, and the IC_50_ was 0.9 μg/ml. The mycelium has a good prospect in applying mycelium meat and the development of antihypertensive peptides. Therefore, the peptides' taste characteristics and pharmacological activities in the fermented mycelia of *S. rugosoannulata* deserve further exploration.

Researchers have isolated and identified the taste peptides from the fruiting bodies of shiitake mushroom (3), *Volvariella volvacea* (4), *Agaricus bisporus* (5), and *S. rugosoannulata* (6). These peptides mainly display the taste characteristics of umami and kokumi in mushroom-fruiting bodies. To avoid the interference of other components in the fruiting body, it is usually necessary to extract, separate, and purify the peptides in the fruiting body. The peptide mixture with a large peak area or better taste in separation fractions would be selected for further analysis. These selected fractions cannot reflect the overwhelming taste characteristics of raw materials.

In recent years, the importance of Peptidomics in food science has been increasing daily. Food Peptidomics technology has successfully identified food's bioactive peptides, taste peptides, and peptide biomarkers. The peptides in the mycelia of *S. rugosoannulata* were identified by the non-targeted analysis method of Peptidomics. Then, the database of peptides was statistically analyzed by bioinformatics to clarify the basis of the characteristic structure of the peptides. Then, the specific peptides were screened for targeted research, which provides technical support for analyzing the taste structure and characteristics of the peptides of *S. rugosoannulata*. The mechanism of taste perception of *S. rugosoannulata* taste peptide can be investigated by studying the binding degree between taste peptide and the sensory receptor and the recognition of taste peptide by the receptor. Molecular docking technology simulates the biological process of molecular interaction, reveals the structural basis of ligand recognition by receptor and its binding with the receptor in receptor-ligand interaction, and predicts the taste perception mechanism of taste peptides at the atomic level. Molecular docking technology has been applied to the binding sites and interaction mechanisms between taste peptides and their taste receptors T1R1/T1R3 (7–10). The molecular docking technique was used to explore the active structure and taste perception mechanism of the taste peptide of *S. rugosoannulata*, combined with the taste receptors T1R1/T1R3, which could provide theoretical support for understanding its pleasant taste.

Therefore, based on the previous research, this study carried out: (I) the amino acid sequence of peptides in the fermentation mycelium of *S. rugosoannulata* was analyzed, and the taste characteristics of peptides were predicted; (II) the proportion of amino acids in the peptide and the characteristic taste structure basis of the taste peptides was revealed; (III) the binding mode and binding sites between T1R1/T1R3 receptors and taste peptides were analyzed, the taste mechanism of peptides was clarified. This study provides theoretical support for understanding the pleasant taste characteristics and taste mechanism of *S. rugosoannulata* mycelium and a reference for the development and application of mycelium.

## Materials and methods

### Materials

Approximately, 10% inoculum of *S. rugosoannulata* (NCBI strain release No. SRR14469700) was fermented in a 30-L fermentation tank (liquid volume, a 25-L optimized medium; culture temperature, 26°C; stirring speed, 100 r/min; ventilation capacity, 25 L/min) for 7 days. The fermentation samples were centrifuged at 8,000 g for 10 min, and the mycelium was collected. After the mycelium was thoroughly washed with distilled water, it was freeze-dried at −70°C for 48 h and collected.

### Peptide sequence and precursor protein analysis

LC-MS/MS identified the peptide sequence in the fermented mycelia of *S. rugosoannulata*. The mycelium samples were desalted by the Millipore ZipTip C18 column and dissolved in a 20-μl dissolving solution (0.1% formic acid, 5% acetonitrile). The samples were centrifuged at 8,000 g for 20 min, and 8-μl supernatant was identified by mass spectrometry. Liquid chromatography mobile Phase A is 0.1% formic acid; mobile Phase B is 0.1% formic acid and 80% acetonitrile. LC-MS/MS setting parameters are shown in Supplementary Table 1. The peptide sequence was analyzed by the Peaks software. The protein sequence of *S. rugosoannulata* was retrieved on NCBI. The identified peptide sequence was compared with the protein sequence of *S. rugosoannulata* to determine the protein precursor that produced the peptide.

### Taste characteristics of peptides analysis

Bio-UWM is a peptide database for online analysis of peptide bioactivity. It supports studying the relationship between peptide molecular structure, a potential sensory profile, and sensory properties. Therefore, BIO-UWM (https://biochemia.uwm.edu.pl/biopep-uwm/) is used to predict the taste characteristics of peptides identified by LC-MS/MS, and the peptides with salty and umami taste characteristics were screened.

### Amino acid distribution of peptides analysis

Seqlogo was used to analyze the amino acid distribution in the salty and umami peptides. The amino acid character represents its proportion. The larger the amino acid character, the higher the amino acid ratio (11).

### Taste mechanism of peptides analysis

The taste mechanism of the peptides from *S. rugosoannulata* was analyzed by molecular docking. Our previous homologous modeling has selected the optimized structure of the T1R1/T1R3 taste receptors. The molecular docking software was used to find the active amino acid sites in the T1R1/T1R3 receptors' binding pocket. The 3D molecular structures of the salty and umami peptides of *S. rugosoannulata* were constructed by software as the docking ligands of the T1R1/T1R3 taste receptors, and the peptides were optimized for energy minimization. The software was used for the semi-flexible docking of T1R1/T1R3 and peptides, and the peptide-receptor binding complexes with low binding energy were screened, and the binding sites and modes of action were analyzed.

### Statistical analysis

Python was used to analyze and plot the molecular weight distribution, MS proportion, and amino acid proportion of LC-MS/MS-identified peptides.

## Results

### Distribution of peptides in the mycelia of *S. rugosoannulata*

**Figure 1:**
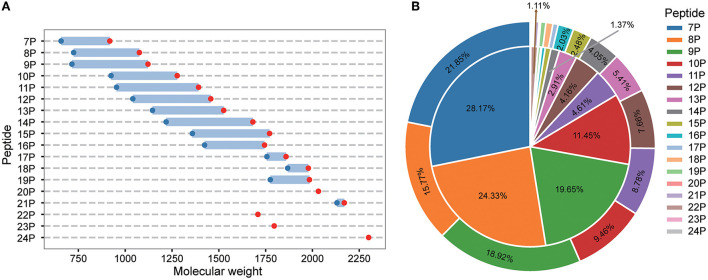
Molecular weight **(A)**, MS area [**(B)**, the inner layer], and number [**(B)**, the outer layer] of peptides in the fermented mycelia of *S. rugosoannulata*. 7P-24P stands for heptapeptides-24 peptides. The unmarked proportion was part of the MS area, and the number was <1%.

LC-MS/MS identified a total of 748 peptides. The identified peptide was compared with the protein sequence of *S. rugosoannulata*, and the protein precursor of 444 peptides could be found. The molecular weight distribution, number, and MS area proportion of 444 types of peptides were analyzed. The statistical results are shown in Figures 1A,B. The molecular weight distribution of the peptides in the fermentation mycelium of *S. rugosoannulata* was 657–2,300 Da (Figure 1A), and the peptide distribution was a heptapeptide-24 peptide (Figure 1B). Heptapeptide to decapeptide accounted for 11.45%−28.17% of the number and 9.46%−21.85% of the MS area in mycelial peptides. Heptapeptides to decapeptides were the main peptides in the fermentation mycelia of *S. rugosoannulata*.

### Taste characteristics of peptides in fermentation mycelia of *S. rugosoannulata*

**Figure 2:**
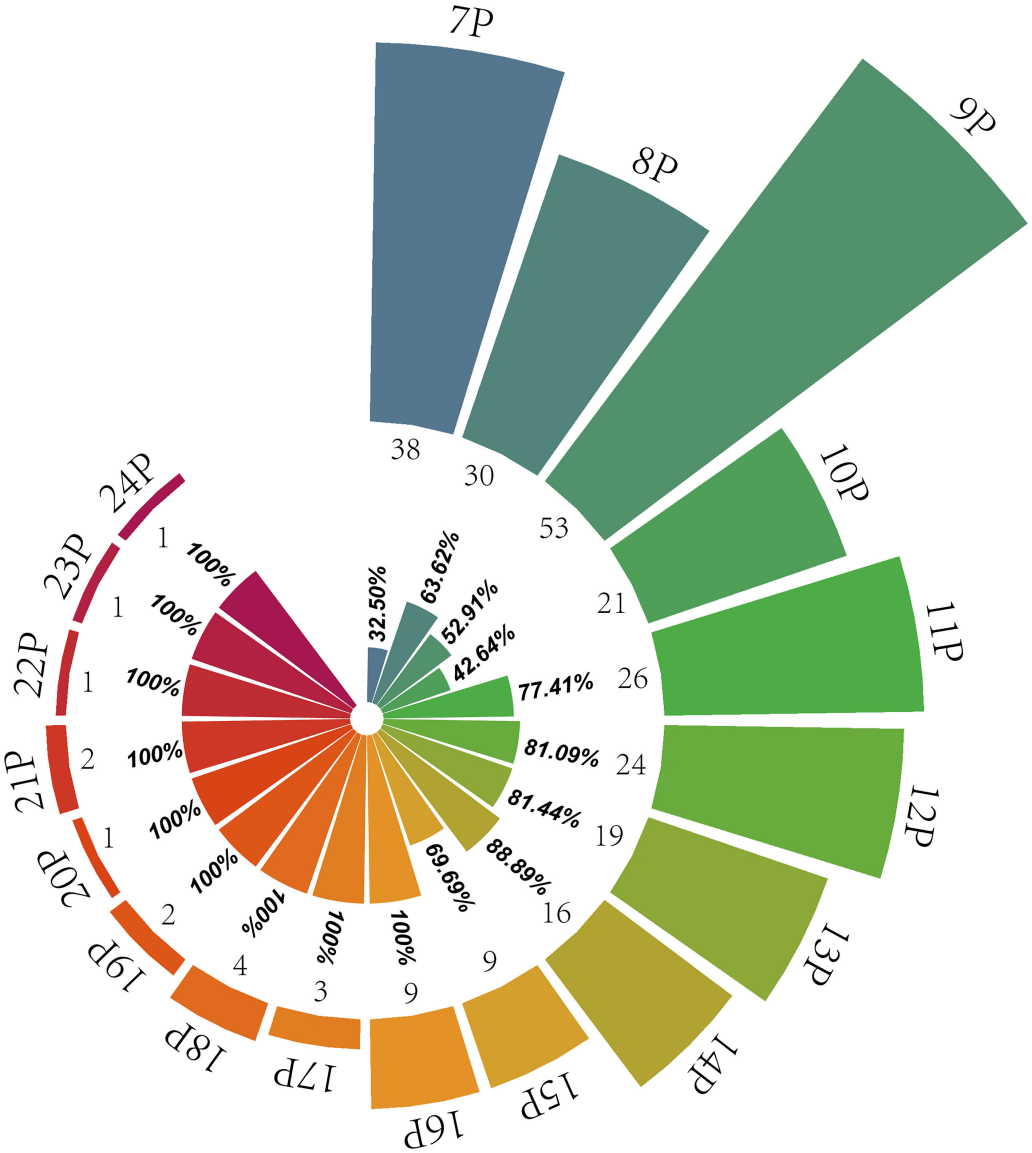
Distribution of pleasant taste peptides in the fermentation mycelium of *S. rugosoannulata*. 7P-24P stands for heptapeptides to 24 peptides; the internal rose chart shows the MS area proportion of salty and umami peptides in their respective peptides (32.50%−100%), and the external rose chart shows the numbers of pleasant taste peptides (1–53).

BIO-UWM was used to predict the taste characteristics of 444 identified peptides. The prediction results show that the number of peptides (260) with salty and umami taste characteristics and MS (53.22%) proportion was high. The statistical results are shown in Figure 2. Most kinds and quantities of taste peptides were nonapeptides, followed by heptapeptides and octapeptides. The MS area proportion of heptapeptides to decapeptides ranged from 32.50 to 63.62%. Among the 11 peptides to 15 peptides, the numbers of peptides with salty and umami taste characteristics were relatively affluent, so the MS area proportions of salty and umami peptides were also high (69.69%−88.89%). Among the hexadecapeptides to 24 long-chain peptides, the numbers of peptides had no advantage, but the identified peptides all had salty and umami taste characteristics (accounting for 100%). It can be seen from the specified numbers and the MS area proportion of undecanoic peptides to 24 peptides that the long-chain peptides also played an essential role in the pleasant taste characteristics of *S. rugosoannulata* mycelium. In conclusion, heptapeptides-decapeptides as the main peptide, undecanoic peptides to 24 peptides as the important taste peptide constituted the characteristic peptides' structural basis of the *S. rugosoannulata* fermentation mycelium.

### Amino acid distribution of peptides in fermentation mycelia of *S. rugosoannulata*

**Figure 3:**
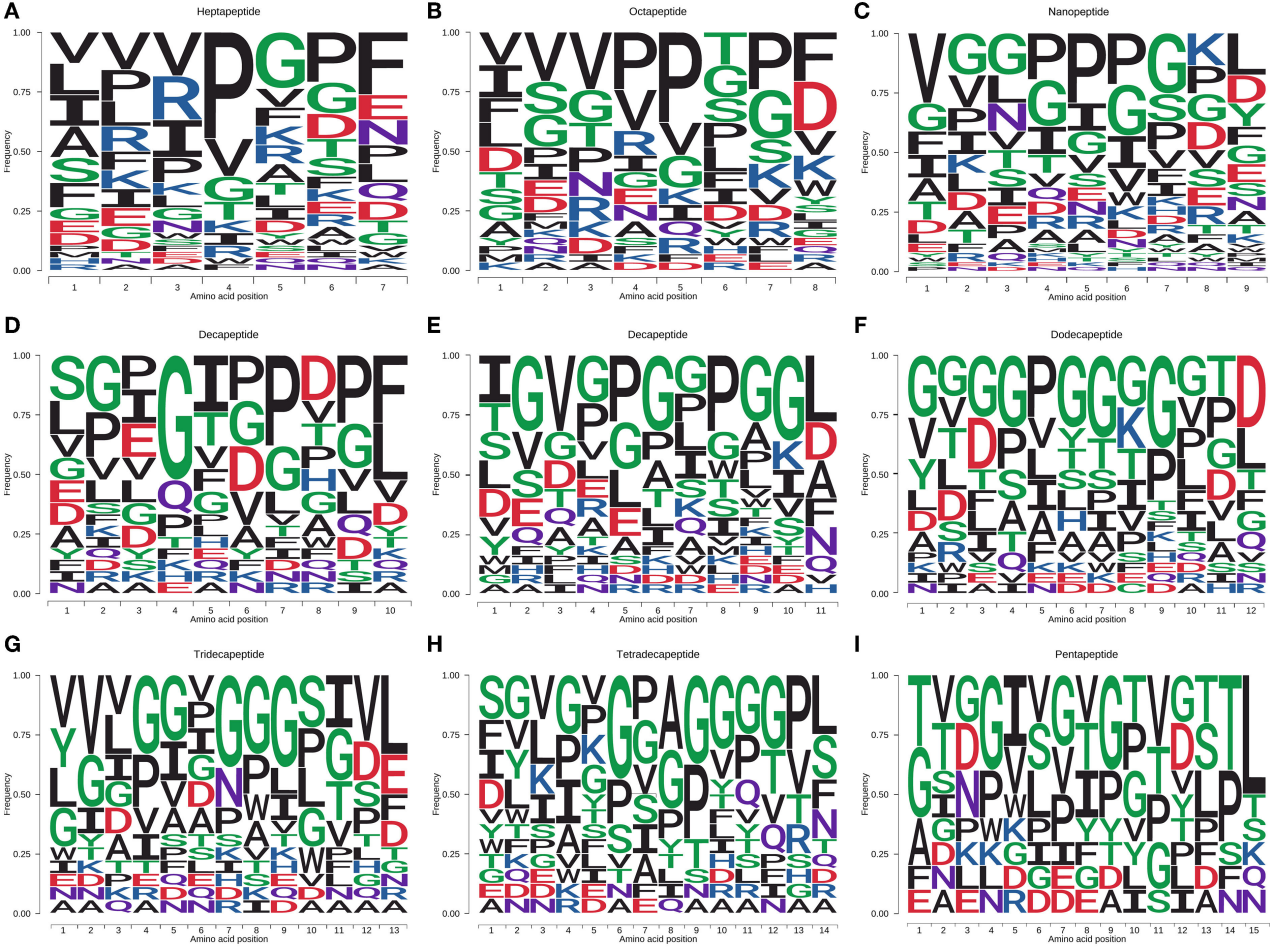
Amino acid distribution of salty and umami peptides [**(A–I)** heptapeptide to pentapeptide] in the fermented mycelium of *S. rugosoannulata*. F, I, L, M, V, W, A, P, hydrophobic amino acids; R, K, H, basic amino acids; D, E, acidic amino acids; G, S, T, C, N, Q, Y, polar-uncharged amino acids.

The amino acid distribution of the peptides with salty and umami taste characteristics was analyzed, and the results are shown in Figures 3A–I. Heptapeptides and octapeptides were similar in amino acid distribution. For example, on the C-terminal and the second position of the C-terminal, the amino acid distribution was mainly F, P, G, and D, and the proportion of V, P, I, R, and G in the peptide was relatively high. V, I, L, S, and F were the primary amino acids at the N-terminal and the second position. E and N were abundant at the C-terminal of heptapeptides; P, A, and R in the second position at the N-terminal and N-terminal of heptapeptides. S and T were plentiful in the octapeptides, and G was generous in the second position at the N-terminal and N-terminal. The number of salty and umami peptides identified by non-apeptides was the most abundant. The extension of the peptide chain and the diversity of peptide types made the distribution of amino acids in peptides more diverse. L in the C-terminal and the second C-terminal of non-apeptides accounted for the highest proportion, followed by K, P, D, G, and Y. The distribution proportion of the last five amino acids had little difference; D and G occupied a certain proportion in the second position at the C-terminal and the C-terminal. In the non-apeptides, the distribution of amino acids was mainly P, G, I, S, and V, which was like that in the heptapeptides and octapeptides. The N-terminal and the second amino acid distribution at the N-terminal was mainly V, G, P, F, and I. In conclusion, among the identified salty and umami peptides of *S. rugosoannulata*, the distribution of non-polar hydrophobic amino acids and polar-uncharged amino acids accounted for a relatively high proportion, and the ratio of polar-uncharged amino acids further increased with the extension of the peptide chain. P, F, I, L, V, G, S, T, and D were the amino acids with a high proportion in the peptides.

### Taste mechanism of peptides in the mycelia of *S. rugosoannulata*

T1R1/T1R3 is a G protein-coupled receptor with a dimer structure and many active sites (12). Researchers (13, 14) constructed a nanogold sensor with receptor T1R1 as the recognition element to investigate the binding constants of different taste substances and found that T1R1 can be used as the essential receptor for taste recognition. Based on molecular docking and sensory evaluation in the early stage, we also verified that T1R3 is the critical receptor for recognizing umami taste (6). Therefore, the taste peptides in the mycelia of *S. rugosoannulata* were docked with T1R1 and T1R3 to reveal their taste mechanism.

**Figure 4:**
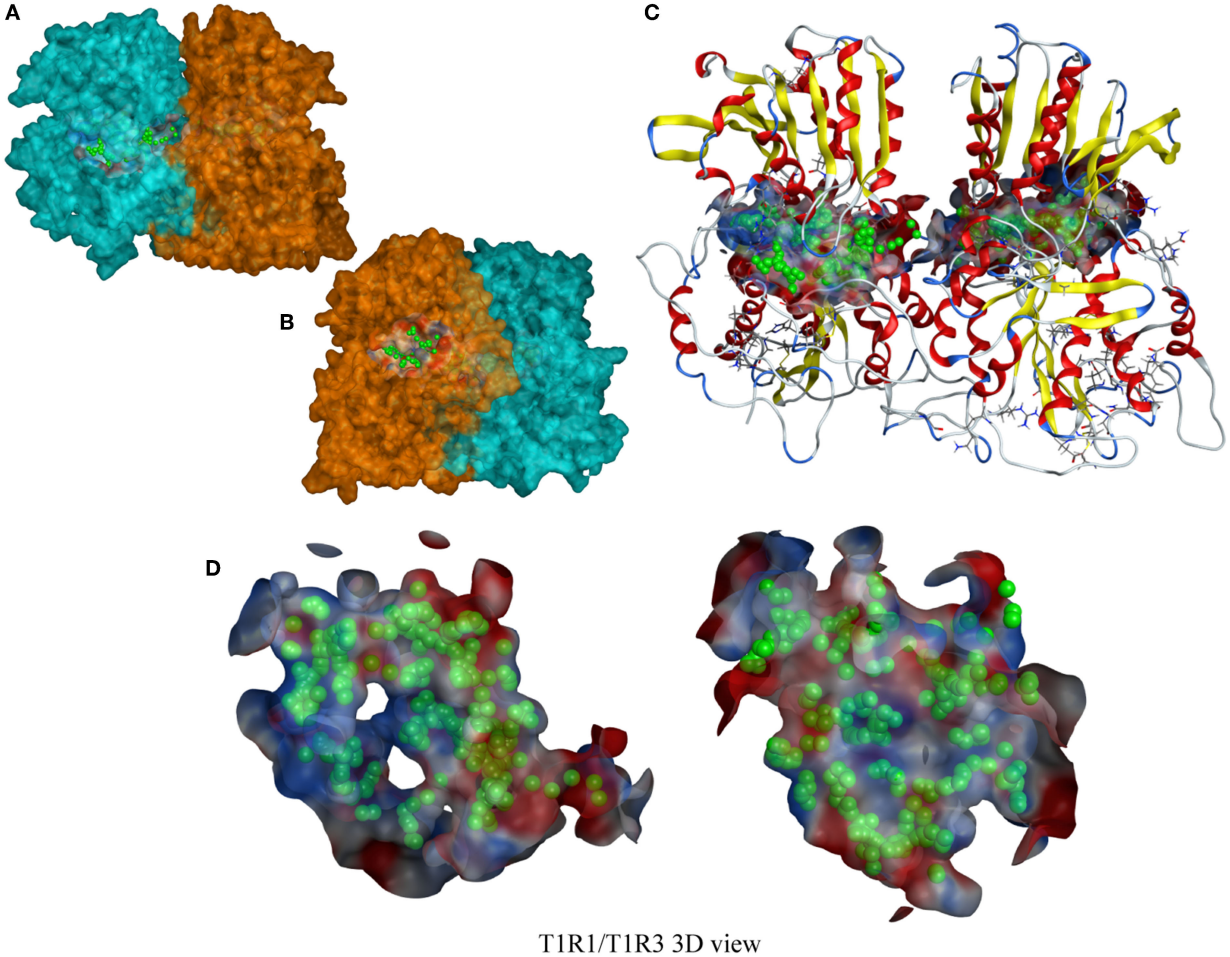
A T1R1/T1R3 3D view. **(A,B)** a T1R1/T1R3 molecular surfaces view, the blue part is T1R1, the orange part is T1R3; **(C)** a T1R1/T1R3 ribbon-style view, including a docking cavity and binding amino acid sites (the green sphere); **(D)** a docking cavity and binding amino acid sites in T1R1/T1R3 receptor.

The T1R1/T1R3 receptor structure is based on our previous construction (6). The cavity-binding domains and amino acid-binding sites of the T1R1/T1R3 receptor predicted by molecular docking are shown in Figures 4A–D. The binding active amino acid sites in the T1R1 cavity-binding domain are 59 amino acid residues. The binding active amino acid sites in the T1R3 cavity-binding domain comprise 55 amino acid residues (Supplementary Table 2).

The docking results between the taste peptides and T1R1/T1R3 show that the peptide can bind to the active amino acid residues in the cavity-binding domains of receptor T1R1/T1R3. Among them, the amino acid residues attached to the T1R1 receptor were mainly Asp147, Asp218, Asp219, Asn150, Ser217, Cys50, Asp108, Ser148, Gly49, Ala170; Glu45, Glu148, Ser104, Asp215, His278, Glu48, Ala46, Arg64, Ala302, and Asn68, which are the critical amino acid residues for the peptides to bind to the TIR3 receptor. Due to the diversity of peptide types, the types of amino acid residues that the peptides can attach to the receptors were also relatively affluent. More than 60% of the active amino acid residues in the cavity-binding domain of the T1R1/T1R3 receptors can form interactions with peptides. The high proportion and interaction force of amino acid residues were critical to the peptide to produce a pleasant taste.

**Figure 5:**
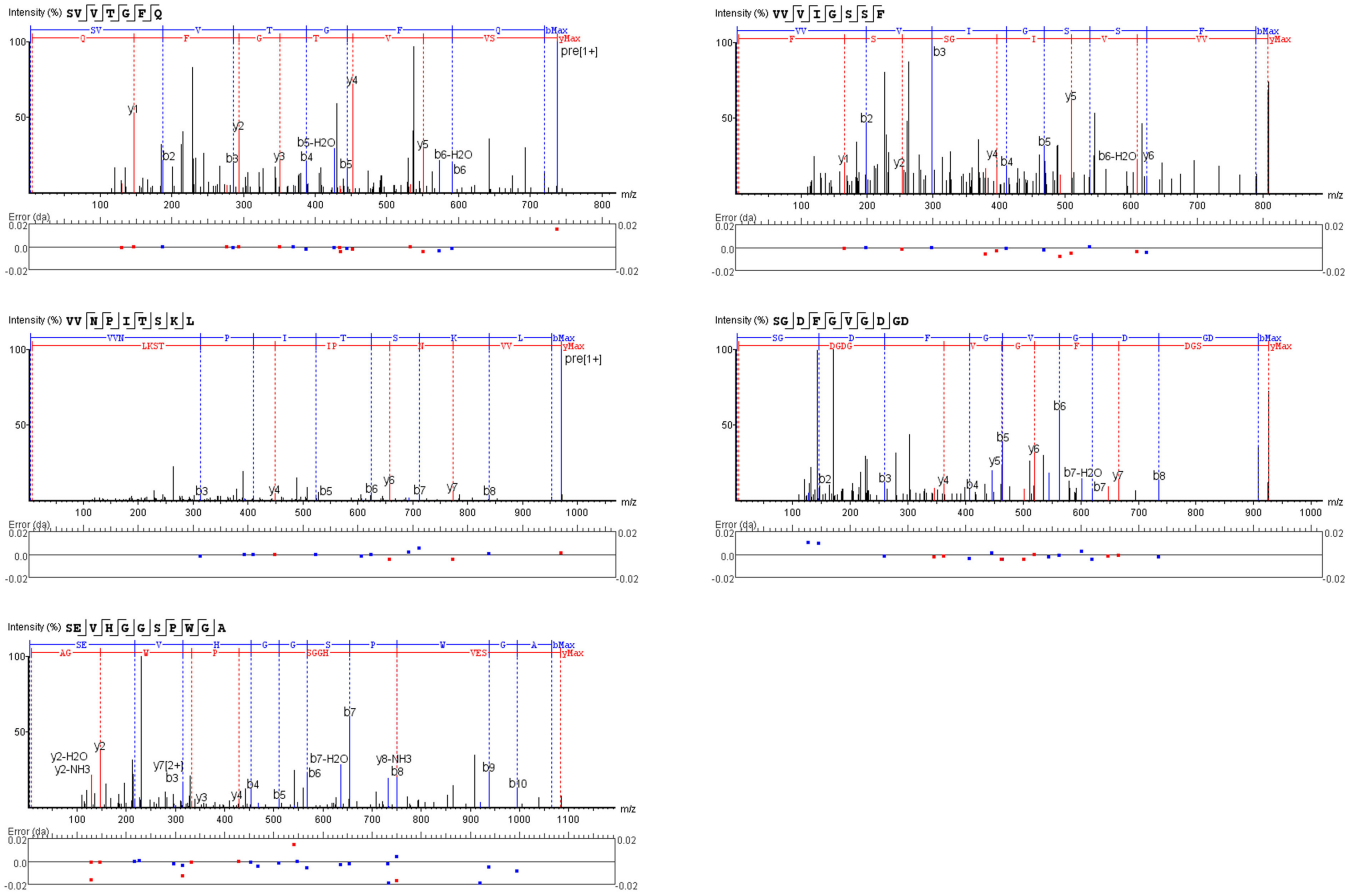
A secondary mass spectrum of peptides.

**Figure 6:**
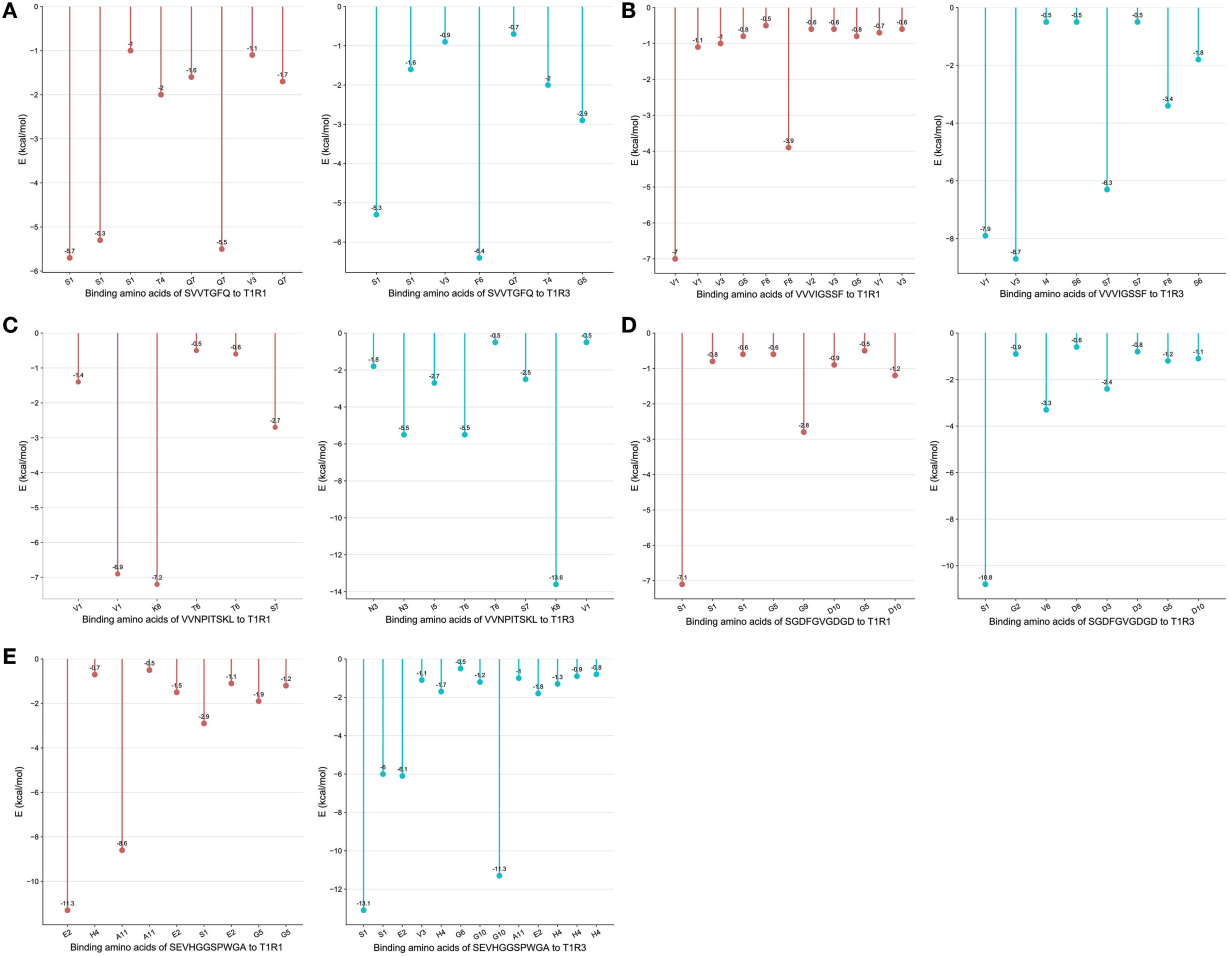
**(A–E)** Binding amino acids of peptides to T1R1/T1R3.

**Figure 7:**
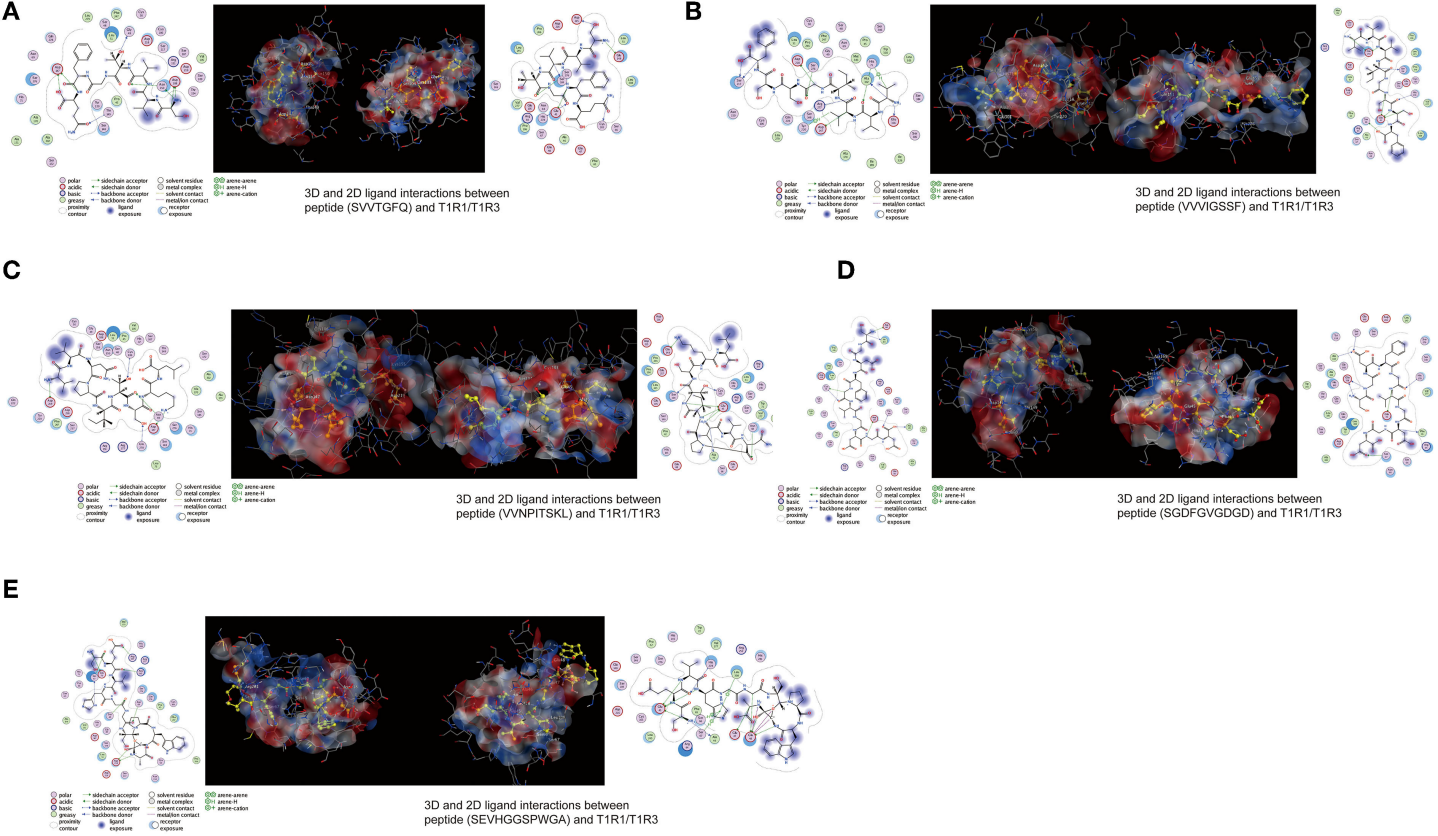
**(A–E)** 3D and 2D ligand interactions between peptides and T1R1/T1R3 amino acids. The left figure shows the 2D diagram of peptide binding to T1R1, the middle figure shows the 3D graph of peptide docking to T1R1/T1R3, including the electrostatic model of the receptor cavity-binding domain, the yellow ball and stick model peptide, and the binding amino acid sites, and the right figure shows the 2D diagram of peptide binding to T1R3.

The peptide with a typical characteristic structure was screened (the peptide identification information is shown in Supplementary Table 3, Figure 5). The docking results of SVVTGFQ, VVVIGSSF, VVNPITSKL, SGDFGVGDGD, and SEVHGGSPWGA with T1R1/T1R3 are shown in Figures 6, 7. The peptides can enter the cavity-binding domain of the T1R1/T1R3 receptors and interact with the active amino acid residues in the cavity-binding domain.

From the docking results, the docking score of SVVTGFQ and T1R1 was −10.54 kcal/mol. Peptide and T1R1 form hydrogen bond interaction (eight hydrogen bonds) and ionic interaction. The peptide's amino acids interacting with T1R1 were S, V, T, and Q. The T1R1 amino acid residue sites to which the peptide was bound include Gly49 and Thr149 of the T1R1 backbone, Asp108, Asn150, and Asp147 of the side chain. It can be seen from the binding energy between each amino acid and the receptor amino acid site that S contributed the most to the binding energy between the peptide and T1R1, followed by Q (Figure 6A). The docking score of SVVTGFQ and T1R3 was −10.83 kcal/mol, and the peptide formed hydrogen bond interaction (seven hydrogen bonds) and ionic interaction with T1R3. The peptide's amino acids interacting with T1R3 were S, V, T, F, G, and Q. The T1R3 amino acid residues to which the peptide was bound to include Asp215, Ser104, Cys103, His278 of the T1R3 backbone, Glu148, Glu45, and Ser276 of the side chain. F contributed the most to the binding energy between the peptide and T1R3, followed by S (Figure 6A). To sum up, S, V, T, and Q, especially S, played a significant role in the docking process of SVVTGFQ with T1R1/T1R3. Asp108, Asn150, Asp147 in T1R1, Glu45, and Glu148 in T1R3 played a substantial role in forming stable receptor-ligand complexes (Figure 7A).

The docking score between VVVIGSSF and T1R1 was −10.86 kcal/mol, and the peptide formed nine hydrogen bond interactions and H-pi interactions with T1R1. The peptide's amino acids that interacted with T1R1 were V, G, and F. V1, V2, and V3 formed hydrogen bond interactions with Glu301, Ala302, Arg277 of the T1R1 backbone and Asp147 and His71 of the T1R1 side chain. G created hydrogen bond interaction with Asp218 of the T1R1 side chain. F formed hydrogen bond interaction with Ser217 of the T1R1 backbone. In addition, 2 H-pi interactions were formed between V1 and receptors 5-ring His71, V3 and receptors 6-ring Tyr220. V1 contributed the most to the binding energy between the peptide and T1R1, followed by F8 (Figure 6B). The docking score between VVVIGSSF and T1R3 was −10.40 kcal/mol. The hydrogen bond formation interaction (nine hydrogen bonds) and ionic interaction were formed between the peptide and T1R3. The amino acids in the peptide that interacted with T1R3 were V, I, S, and F. The peptide interacted with Ser104, His278, Glu148, Glu45, and Ser66 of the T1R3 backbone to form hydrogen bonds. V3 contributed the most to the binding energy between the peptide and T1R3, followed by V1, S7, and F8 (Figure 6B). In conclusion, V played a significant role in the docking process of VVVIGSSF with T1R1/T1R3, especially V1. The amino acids V, S, and F in the peptide, Glu301 in T1R1, Glu45, and Ser66 in T1R3 significantly formed stable receptor-ligand complexes (Figure 7B).

The docking score of VVNPITSKL and T1R1 was −12.53 kcal/mol. Peptide and T1R1 formed hydrogen bond interaction (six hydrogen bonds) and ionic interaction. The amino acids that interacted with T1R1 in the peptide were V, T, S, and K. The peptide interacted with Cys106, Gln278 of the T1R1 backbone, Asp219, Asp147, and Asn69 of the side chain to form hydrogen bonds. K contributed the most to the binding energy between the peptide and T1R1, followed by V1 (Figure 6C). The docking score of VVNPITSKL and T1R3 was −10.70 kcal/mol. Peptide and T1R3 developed hydrogen bond interaction (nine hydrogen bonds) and ionic interaction. The amino acids in the peptide that interacted with T1R3 were V, N, I, T, S, and K. The peptide interacted with Cys103, Ser104 of the T1R3 backbone, Glu45, Ala46, Glu148, and Asn68 of the side chain. K contributed the most to the binding energy between the peptide and T1R3, followed by N and T (Figure 6C). In conclusion, V, T, S, and K played a significant role in the docking process of VVNPITSKL with T1R1/T1R3, especially K. Asp147 and Asp219 in T1R1, Glu148 and Glu45 in T1R3 played a substantial role in the formation of stable receptor-ligand complexes (Figure 7C).

The score of SGDFGVGDGD docking with T1R1 was −11.45 kcal/mol. Peptide and T1R1 formed hydrogen bond interaction (eight hydrogen bonds) and ionic interaction. The amino acids that interacted with T1R1 were S, G, and D. The peptide interacted with Ile244, Cys50, Thr149 of the T1R1 backbone, and Asp243, Cys50, Asp147, and Ala170 of the side chain to form hydrogen bonds. The score of SGDFGVGDGD docking with T1R3 was −12.86 kcal/mol. The peptide interacted with T1R3 to form nine hydrogen bonds. The amino acid residues in the peptide that interacted with T1R3 were S, G, D, and V. The peptide interacted with Glu301, Phe65, Ser147, Ala169, His278, Glu45, and Ser67 of the T1R3 backbone to form hydrogen bonds. S contributes the most to the binding energy between the peptide and T1R1/T1R3 (Figure 6D). In conclusion, S, G, and D played a significant role in the docking process of SGDFGVGDGD with T1R1/T1R3, especially S. Asp243 in T1R1 and Glu301 in T1R3 played a substantial role in the formation of stable receptor-ligand complexes (Figure 7D).

The score of connection between SEVHGGSPWGA and T1R1 was −9.69 kcal/mol. Peptide and T1R1 formed hydrogen bond interaction (nine hydrogen bonds) and ionic interaction. The amino acid residues in the peptide that interacted with T1R1 were S, E, H, G, and A. The peptide interacted with Ser67 of the T1R1 backbone, Glu70, Asp218, Ser67, Arg307, Arg281, Ser48, and Asn69 of the side chain to form hydrogen bonds. E contributed the most to the binding energy between the peptide and T1R1, followed by A (Figure 6E). The score of connection between SEVHGGSPWGA and T1R3 was −11.72 kcal/mol. Peptide and T1R3 formed hydrogen bond interaction (11 hydrogen bonds), ionic interaction, and pi-H interaction. The peptide's amino acids interacting with T1R3 were S, E, V, H, G, and A. The peptide interacted with Ala46, His278, Glu45, Glu47, and Glu48 of the T1R3 backbone; it formed pi-H interaction with T1R3 5-ring Ser66, Ser67, and Leu308. S contributed the most to the binding energy between the peptide and T1R3, followed by G10 and E (Figure 6E). H in the peptide was the amino acid residue that interacted most with the T1R3 receptor, including one hydrogen bond interaction and three pi-H interactions. To sum up, S, E, H, G, and A played a vital role in the docking process of SEVHGGSPWGA with T1R1/T1R3. E and S at the N-terminal, G and A at the C-terminal of the peptide, Glu70 and Asp218 in T1R1, Glu45, Glu48, and Ala46 in T1R3 played a significant role in stabilizing the receptor-ligand complex (Figure 7E).

In conclusion, the first and second amino acid residues at the C-terminal and N-terminal of the peptide were easy to bind to T1R1/T1R3 receptors, such as S, V, E, K, G, and A, S, V, and K were the key binding amino acid residues in peptides. Undecanoic peptides had more amino acid-binding sites. The taste advantage of the long-chain peptide was the main reason for the interaction between multiple amino acid residues and receptors. Among the peptide-binding sites of T1R1/T1R3 receptors, Asp108, Asn150, Asp147, Glu301, Asp219, Asp243, Glu70, Asp218 in T1R1 and Glu45, Glu148, Glu301, Glu48, and Ala46 in T1R3 were the primary amino acid residues for peptide binding. Hydrogen bond interaction was the primary mode of interaction between peptides and receptors and played an essential role in the receptor's perception of the taste characteristics of the peptide.

## Discussion

According to the literature reports, among the identified food-derived taste short peptides, the taste of peptides mainly depended on the original taste of the constituent amino acids. The peptides formed by the connection of glutamic acid, aspartic acid, serine, glycine, and glutamine had a noticeable umami taste (15, 16). As more and more peptides were isolated and identified, researchers found that more long-chain peptides and more amino acids played an essential role in food taste (7, 9, 17–25).

Yu et al. (26) isolated and purified four umami peptides (VPY, TAY, AAPY, and GFP). It was found that the peptide contained bitter amino acids instead of umami amino acids. In addition, the taste of peptides was better than their amino acids, which indicated that the taste of peptides did not depend entirely on their amino acids. Similarly, Xu et al. (4) identified the umami peptides ASNMSDL and LQPLNAH in *V. volvacea*; Zhu et al. (8) extracted the umami hexapeptide INKPGL, SDSCIR, and GPDPER from the myosin of Atlantic cod (*Gadus morhua*); and the types of amino acids in these peptides were also more abundant (9, 20). It was also found that hydrophobic amino acids accounted for a high proportion of umami peptides, and some hydrophobic amino acids had a vital contribution to their umami taste (27). In this study, the balance of polar-uncharged amino acids S and G and hydrophobic amino acids in the taste peptides identified from the fermentation mycelium of *S. rugosoannulata* were higher. Therefore, the contribution of polar amino acids and hydrophobic amino acids to the taste of the mycelia peptide of *S. rugosoannulata* should not be ignored.

The sensing mechanism of taste peptides has also attracted much attention. The molecular weight distribution, amino acid composition, and spatial structure of peptides have an essential impact on the receptor sensing of peptides. The interaction between peptide and receptor affects the subsequent intracellular taste perception signal transduction and brain response (28). It was found that T1R1-VFT was a potential binding site (9, 10, 25) for taste peptides; At the same time, some studies revealed that the taste peptides were mainly bound to T1R3-VFT (7, 8, 20, 23, 29, 30).

Liang et al. (31) isolated and identified hexapeptide and heptapeptide from chicken soup. The results showed that His71, Ser107, and Glu301 of T1R1 and Asp216, Ser104, His145, and Ala302 of T1R3 were the key binding sites of peptide receptor interaction. Yang et al. (29) found that the peptide identified from mandarin fish interacted with Ser, Glu, His, Gln, Arg, and Lys residues in T1R3. Yu et al. (10) identified umami peptides from myosin. Through molecular docking, it was determined that the primary amino acid residues of the peptide binding to T1R1 were Arg151, Asp147, and Gln52. Deng et al. (32) isolated five peptides from *Trachinotus ovatus* hydrolysate. Asp192 and Glu301 were the primary amino acid-binding sites in T1R3. The peptides isolated from tilapia lower jaw by Ruan et al. (22) were mainly combined with Glu148 and other amino acid residues of T1R3. His71, Asp147, Glu301 in T1R1, Ser104, Ala302, and Glu148 in TIR3 were the critical active amino acid residues for the interaction between the peptides and T1R1/T1R3 receptors. Therefore, although the identified peptides were different, as reported by researchers, identical receptor amino acid residues still played a significant role in peptide receptor interaction.

**Figure 8:**
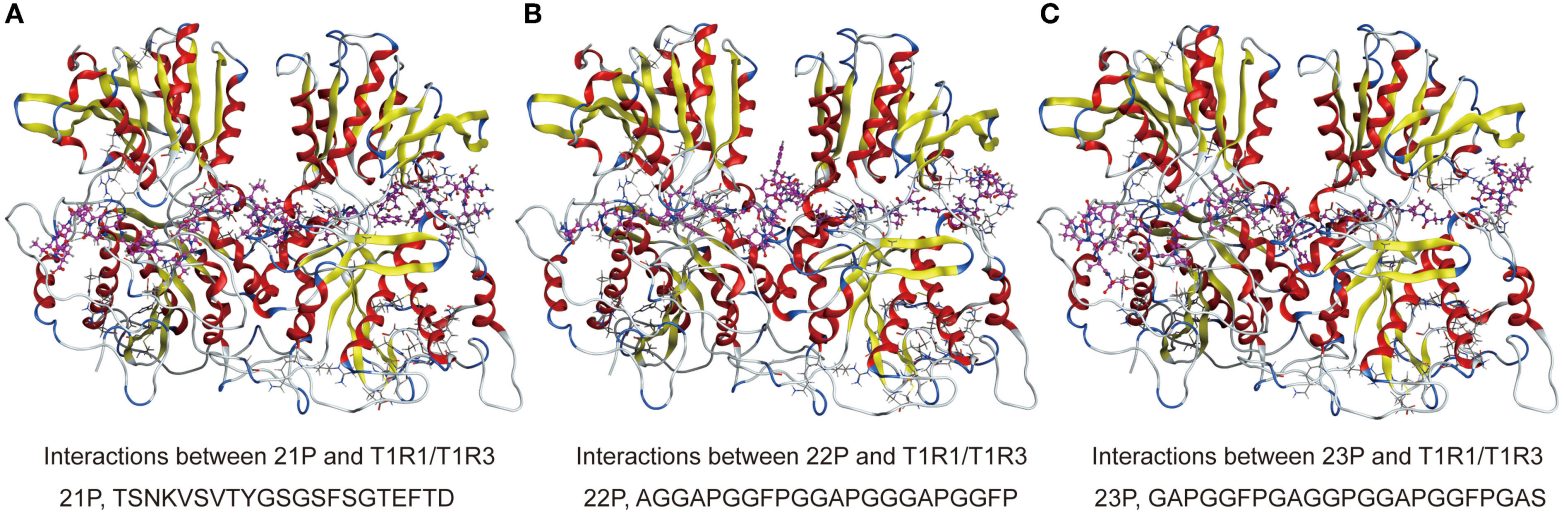
**(A–C)** 3D ligand interactions between peptides and T1R1/T1R3 amino acids. T1R1/T1R3, the cartoon model; peptides, the purple ball and the stick model.

The peptide of *S. rugosoannulata* in this study can bind to the active amino acid sites of the T1R1/T1R3 receptor cavity, indicating that the peptide structure of *S. rugosoannulata* has advantages in interaction with the receptor. As shown in Figures 8A–C, the long-chain peptides can partially enter the receptor cavity and interact with the receptor active amino acid sites (21P, 22P, 23P, purple bat model peptides). 24P was not docked due to acetylation modification. Amin et al. (25) found that GENEEEDSGAIVTVK was connected to the surface of T1R1 and could not enter the receptor cavity, mainly related to the 3D structure of the peptide.

The binding sites and the four interaction forces (including electrostatic interaction, hydrogen bond interaction, van der Waals interaction, and hydrophobic interaction) involved in the interaction between taste peptides and receptors were discussed. The results may vary due to different docking methods and software, but hydrogen bonds were still the main force (7, 22, 29, 30). It was found that ligand binding can regulate the size of the receptor cavity-binding pocket (33). S and G played a vital role in the binding and interaction between the peptides of *S. rugosoannulata* and T1R1/T1R3 receptors. At the same time, the peptides identified in this study can form interactions with T1R1 and T1R3, respectively. Surface plasmon resonance and other technologies can further explore the tendency and selection of peptides binding with the receptors, which has important reference significance for understanding the taste characteristics of the peptides in the fermented mycelium of *S. rugosoannulata*.

In recent years, scholars have gradually explored the enzymatic hydrolysis process and Maillard reaction of the taste peptide composite matrix to accelerate the application research of taste peptides. Taste peptides usually synergize with other taste substances (34). In the mycelia of *S. rugosoannulata*, the peptide accounted for a relatively high proportion. Its long-chain peptides have pleasant taste characteristics, so it is unnecessary to extract a single taste peptide, which can be directly used as raw material to produce flavor-based material.

In this study, the docking results between long-chain peptides and T1R1/T1R3 were still good, which were not related to chain length but were more related to the 3D structures of the peptides. Long-chain peptides can partially or entirely enter the receptor cavity, interact with the active amino acid sites in the receptor cavity, and then exert pleasant taste characteristics. At the same time, the docking results showed that the long-chain peptide exposed more peptide amino acid residues that could bind to the receptor and then be connected to more receptor cavity active amino acid sites. S and G docking to the receptor played an essential role in the taste presentation of the peptide. The molecular weight of the identified mycelia peptide of *S. rugosoannulata* was <3,000 Da, which was consistent with the reported taste amino acid molecular weight of <3,000 Da, which was also the reason for the pleasant taste of mycelia.

## Conclusion

The molecular weight of the peptides in the *S. rugosoannulata* mycelium was below 3,000 Da. Heptapeptides to decapeptides were the main peptides. Oligopeptides as the main peptides and long-chain peptides as the important taste peptides constituted the structural basis of the characteristic peptides of the mycelia of *S. rugosoannulata*. P, F, I, L, V, G, S, T, and D were the amino acids with a high proportion in the peptide. The peptides can bind with more than 60% of the active amino acid residues in the cavity-binding domain of the T1R1/T1R3 receptors to form hydrogen bond interaction, iron interaction, and pi-H interaction. Among them, hydrogen bond interaction was the primary mode of action between the peptides and the receptors and played an essential role in the receptor's perception of the taste characteristics of the peptides. The first and second amino acid residues at the C-terminal and N-terminal of the peptides were easy to bind to T1R1/T1R3 receptors, such as S, V, E, K, G, A. Asp108, Asn150, Asp147, Glu301, Asp219, Asp243, Glu70, and Asp218 in T1R1 and Glu45, Glu148, Glu301, Glu48, and Ala46 in TIR3 were the key active sites for peptides binding to T1R1/T1R3 receptors.

## Data availability statement

The original contributions presented in the study are included in the article/Supplementary material, further inquiries can be directed to the corresponding author.

## Author contributions

WL: conceptualization, data curation, formal analysis, and writing—original draft. WC: conceptualization, software, and validation. DW and ZZ: methodology and validation. YY: funding acquisition and supervision. All authors contributed to the article and approved the submitted version.

## Funding

This research was funded by Shanghai Agriculture Applied Technology Development Program, China (No. 2020-02-08-00-12-F01484), National Natural Science Foundation of China (No. 31901812), and SAAS Program for Excellent Research Team (No. G2022003).

## Conflict of interest

The authors declare that the research was conducted in the absence of any commercial or financial relationships that could be construed as a potential conflict of interest.

## Publisher's note

All claims expressed in this article are solely those of the authors and do not necessarily represent those of their affiliated organizations, or those of the publisher, the editors and the reviewers. Any product that may be evaluated in this article, or claim that may be made by its manufacturer, is not guaranteed or endorsed by the publisher.

## References

[1] Dudekula UT Doriya K Devarai SK. A critical review on submerged production of mushroom and their bioactive metabolites. 3 Biotech. (2020) 10:337. 10.1007/s13205-020-02333-y 32670737 PMC7343686

[2] Li W Feng J Ma HL Chen WC Wu D Zhang Z. Analysis of the characteristic flavor components and flavor characteristics of the fermentation of *Stropharia rugoso-annulata* based on targeted metabolite assay. J Food Saf Qual. (2022) 13:2736–44. 10.19812/j.cnki.jfsq11-5956/ts.2022.09.007

[3] Kong Y Zhang LL Zhao J Zhang YY Sun BG Chen HT. Isolation and identification of the umami peptides from shiitake mushroom by consecutive chromatography and LC-Q-TOF-MS. Food Res Int. (2019) 121:463–70. 10.1016/j.foodres.2018.11.060 31108770

[4] Xu XD Xu R Song Z Jia Q Feng T Huang MG. Identification of umami-tasting peptides from *Volvariellavolvacea* using ultra performance liquid chromatography quadrupole time-of-flight mass spectrometry and sensory-guided separation techniques. J Chromatogr A. (2019) 1596:96–103. 10.1016/j.chroma.2019.03.003 30871753

[5] Feng T Wu Y Zhang ZW Song S Zhuang HN Xu ZM. Purification, identification, and sensory evaluation of Kokumi peptides from *Agaricusbisporus* mushroom. Foods. (2019) 8:43. 10.3390/foods8020043 30699948 PMC6406481

[6] Chen WC Li W Wu D Zhang Z Chen H Zhang JJ. Characterization of novel umami-active peptides from *Stropharia rugoso-annulata*mushroom and *in silico* study on action mechanism. J Food Compos Anal. (2020) 110:104530. 10.1016/j.jfca.2022.104530

[7] Bu Y Liu YN Luan HW Zhu WH Li XP Li JR. Characterization and structure-activity relationship of novel umami peptides isolated from Thai fish sauce. Food Funct. (2021) 12:5027–37. 10.1039/D0FO03326J 33955998

[8] Zhu WH He W Wang F Bu Y Li XP Li JR. Prediction, molecular docking and identification of novel umami hexapeptides derived from Atlantic cod (*Gadus morhua*). Int J Food Sci Tech. (2020) 56:402–12. 10.1111/ijfs.14655

[9] Wang WL Yang L Ning MH Liu ZY Liu Y. A rational tool for the umami evaluation of peptides based on multi-techniques. Food Chem. (2022) 371:131105. 10.1016/j.foodchem.2021.131105 34537606

[10] Yu ZP Kang LX Zhao WZ Wu SJ Ding L Zheng FP. Identification of novel umami peptides from myosin via homology modeling and molecular docking. Food Chem. (2022) 344:128728. 10.1016/j.foodchem.2020.128728 33272753

[11] Fu Y Liu J Hansen ET Bredi WLP Lametsch R. Structural characteristics of low bitter and high umami protein hydrolysates prepared from bovine muscle and porcine plasma. Food Chem. (2018) 257:163–71. 10.1016/j.foodchem.2018.02.159 29622194

[12] Nuemket N Yasui N Atsumi N Yamashita A. Crystal structure of the medaka fish taste receptor T1r2a-T1r3 ligand binding domains in complex with L-alanine. Nat Commun. (2017) 8:15530. 10.2210/pdb5x2n/pdb28534491

[13] Huang YL Lu DQ Liu H Liu SY Jiang S Pang GC. Preliminary research on the receptor-ligand recognition mechanism of umami by an hT1R1 biosensor. Food Funct. (2019) 10:1280–7. 10.1039/C8FO02522C 30801094

[14] Li JS Wang WL Liu J Li H Zhang NL Yang FZ. Human-like performance umami electrochemical biosensor by utilizing co-electrodeposition of ligand binding domain T1R1-VFT and Prussian blue. Food Chem. (2021) 193:113627. 10.1016/j.bios.2021.113627 34534889

[15] Zhang JA Sun-Waterhouse D Su GW Zhao MM. New insight into umami receptor, umami/umami-enhancing peptides and their derivatives: a review. Trends Food Sci Tech. (2019) 88:429–38. 10.1016/j.tifs.2019.04.008

[16] Zhao YG Zhang M Devahastin S Liu YP. Progresses on processing methods of umami substances: a review. Trends Food Sci Tech. (2019) 93:125–35. 10.1016/j.tifs.2019.09.012

[17] Dang YL Gao XC Ma FM Wu XQ. Comparison of umami taste peptides in water-soluble extractions of Jinhua and Parma hams. LWT-Food Sci Technol. (2015) 60:1179–86. 10.1016/j.lwt.2014.09.014

[18] Dang YL Hao L Zhou TY Cao JX Sun YY Pan DD. Establishment of new assessment method for the synergistic effect between umami peptides and monosodium glutamate using electronic tongue. Food Res Int. (2019) 121:20–7. 10.1016/j.foodres.2019.03.001 31108741

[19] Zhuang MZ Lin LZ Zhao MM Dong Y Sun-Waterhouse D Chen HP. Sequence, taste and umami-enhancing effect of the peptides separated from soy sauce. Food Chem. (2016) 206:174–81. 10.1016/j.foodchem.2016.03.058 27041313

[20] Zhang Y Gao XC Pan DD Zhang ZG Zhou TQ Dang YL. Isolation, characterization and molecular docking of novel umami and umami-enhancing peptides from *Ruditapes philippinarum*. Food Chem. (2021) 343:128522. 10.1016/j.foodchem.2020.128522 33208237

[21] Chen MD Gao XC Pan DD Xu SL Zhang HH Sun YY. (2021). Taste characteristics and umami mechanism of novel umami peptides and umami-enhancing peptides isolated from the hydrolysates of Sanhuang Chicken. Eur Food Res Technol. (2021) 247:1633–44. 10.1007/s00217-021-03734-w

[22] Ruan SY Sun LP Sun XD He JL Zhuang YL. Novel umami peptides from tilapia lower jaw and molecular docking to the taste receptor T1R1/T1R3. Food Chem. (2021) 362:130249. 10.1016/j.foodchem.2021.130249 34111693

[23] Li XP Xie XX Wang JX Xu YX Yi SM Zhu WH. Identification, taste characteristics and molecular docking study of novel umami peptides derived from the aqueous extract of the clam *Meretrix meretrix* Linnaeus. Food Chem. (2020) 312:126053. 10.1016/j.foodchem.2019.126053 31884298

[24] Zhang JA Zhao MM Su GW Lin LZ. Identification and taste characteristics of novel umami and umami enhancing peptides separated from peanut protein isolate hydrolysate by consecutive chromatography and UPLC–ESI–QTOF–MS/MS. Food Chem. (2019) 278:674–82. 10.1016/j.foodchem.2018.11.114 30583429

[25] Amin MNG Kusnadi J Hsu JL Doerksen RJ Huang TC. Identification of a novel umami peptide in tempeh (Indonesian fermented soybean) and its binding mechanism to the umami receptor T1R. Food Chem. (2020) 333:127411. 10.1016/j.foodchem.2020.127411 32682228

[26] Yu ZL Jiang HR Guo RC Yang B You G Zhao MM. Taste, umami-enhance effect and amino acid sequence of peptides separated from silkworm pupa hydrolysate. Food Res Int. (2018) 108:144–50. 10.1016/j.foodres.2018.02.047 29735043

[27] Yu XQ Zhang LJ Miao XD Li YY Liu Y. The structure features of umami hexapeptides for the T1R1/T1R3 receptor. Food Chem. (2017) 221:599–605. 10.1016/j.foodchem.2016.11.133 27979247

[28] Wu B Eldeghaidy S Ayed C Fisk ID Hewson L Liu Y. Mechanisms of umami taste perception: from molecular level to brain imaging. Crit Rev Food Sci Nutr. (2021) 1–10. 10.1080/10408398.2021.1909532 33998842

[29] Yang DQ Li CS Li LH Chen SJ Hu X Xiang H. Taste mechanism of umami peptides from Chinese traditional fermented fish (Chouguiyu) based on molecular docking using umami receptor T1R1/T1R3. Food Chem. (2022) 389:133019. 10.1016/j.foodchem.2022.133019 35504076

[30] Dang YL Hao L Cao JX Sun YY Zeng XQ Wu Z. Molecular docking and simulation of the synergistic effect between umami peptides, monosodium glutamate and taste receptor T1R1/T1R3. Food Chem. (2019) 271:697–706. 10.1016/j.foodchem.2018.08.001 30236733

[31] Liang L Duan W Zhang JC Huang Y Zhang YY Sun BG. Characterization and molecular docking study of taste peptides from chicken soup by sensory analysis combined with nano-LC-Q-TOF-MS/MS. Food Chem. (2022) 383:132455. 10.1016/j.foodchem.2022.132455 35183965

[32] Deng XF Lin H Ahmed I Sui JX. Isolation and identification of the umami peptides from *Trachinotu sovatus* hydrolysate by consecutive chromatography and Nano-HPLC-MS/MS. LWT-Food Sci Technol. (2021) 141:110887. 10.1016/j.lwt.2021.110887

[33] Liu H Da LT Liu Y. Understanding the molecular mechanism of umami recognition by T1R1-T1R3 using molecular dynamics simulations. Biochem Biophys Res Commun. (2019) 514:967–73. 10.1016/j.bbrc.2019.05.066 31092329

[34] Wang WL Zhou XR Liu Y. Characterization and evaluation of umami taste: a review. Trac-Trend Anal Chem. (2020) 127:115876. 10.1016/j.trac.2020.115876

